# Maternal serum PlGF associates with 3D power doppler ultrasound markers of utero-placental vascular development in the first trimester: the rotterdam periconception cohort

**DOI:** 10.1007/s10456-024-09939-3

**Published:** 2024-08-14

**Authors:** Eline S. de Vos, A. H. Jan Danser, Anton H. J. Koning, Sten P. Willemsen, Lotte E. van der Meeren, Eric. A. P. Steegers, Régine P. M. Steegers-Theunissen, Annemarie G. M. G. J. Mulders

**Affiliations:** 1 Department of Obstetrics and Gynecology, Erasmus MC University Medical Centre, PO Box 2040, 3000 CA Rotterdam, The Netherlands; 2 Department of Pharmacology, Erasmus MC University Medical Centre, PO Box 2040, 3000 CA Rotterdam, The Netherlands; 3 Department of Pathology, Erasmus MC University Medical Centre, PO Box 2040, 3000 CA Rotterdam, The Netherlands; 4 Department of Biostatistics, Erasmus MC University Medical Centre, PO Box 2040, 3000 CA Rotterdam, The Netherlands

**Keywords:** Placenta, Angiogenesis, Spiral artery remodeling, Angiogenic factors, sFlt-1, Preeclampsia

## Abstract

**Objective (s):**

Circulating angiogenic factors are used for prediction of placenta-related complications, but their associations with first-trimester placental development is unknown. This study investigates associations between maternal angiogenic factors and utero-placental vascular volume (uPVV) and utero-placental vascular skeleton (uPVS) as novel imaging markers of volumetric and morphologic (branching) development of the first-trimester utero-placental vasculature.

**Methods:**

In 185 ongoing pregnancies from the VIRTUAL Placenta study, a subcohort of the ongoing prospective Rotterdam Periconception cohort, three-dimensional power Doppler ultrasounds of the placenta were obtained at 7–9–11 weeks gestational age (GA). The uPVV was measured as a parameter of volumetric development and reported the vascular quantity in cm^3^. The uPVS was generated as a parameter of morphologic (branching) development and reported the number of end-, bifurcation- crossing- or vessel points and total vascular length. At 11 weeks GA, maternal serum biomarkers suggested to reflect placental (vascular) development were assessed: placental growth factor (PlGF), soluble fms-like tyrosine kinase-1 (sFlt-1) and soluble endoglin (sEng). sFlt-1/PlGF and sEng/PlGF ratios were calculated. Multivariable linear regression with adjustments was used to estimate associations between serum biomarkers and uPVV and uPVS trajectories.

**Results:**

Serum PlGF was positively associated with uPVV and uPVS development (uPVV: β = 0.39, 95% CI = 0.15;0.64; bifurcation points: β = 4.64, 95% CI = 0.04;9.25; crossing points: β = 4.01, 95% CI = 0.65;7.37; total vascular length: β = 13.33, 95% CI = 3.09;23.58, all p-values < 0.05). sEng/PlGF ratio was negatively associated with uPVV and uPVS development. We observed no associations between sFlt-1, sEng or sFlt-1/PlGF ratio and uPVV and uPVS development.

**Conclusion(s):**

Higher first-trimester maternal serum PlGF concentration is associated with increased first-trimester utero-placental vascular development as reflected by uPVV and uPVS.

* Clinical trial registration number* Dutch Trial Register NTR6854.

## Introduction

The placenta plays a critical role in health and disease, not only during pregnancy, but also later in life [1]. Healthy placental development and function are highly dependent on adequate establishment of the utero-placental vasculature in early pregnancy [2]. Aberrant vascular development is associated with placenta-related complications, including fetal growth restriction (FGR), preeclampsia (PE) and preterm birth (PTB) [3, 4]. To secure adequate development of the utero-placental vasculature, the maternal uterine vasculature undergoes a variety of adaptations, including formation, dilatation and funneling of spiral arteries [5]. Consequently, markers that monitor development of the utero-placental vasculature in early pregnancy could provide unique insights in the pathophysiology of placental development and placenta-related complications.

**Fig. 1:**
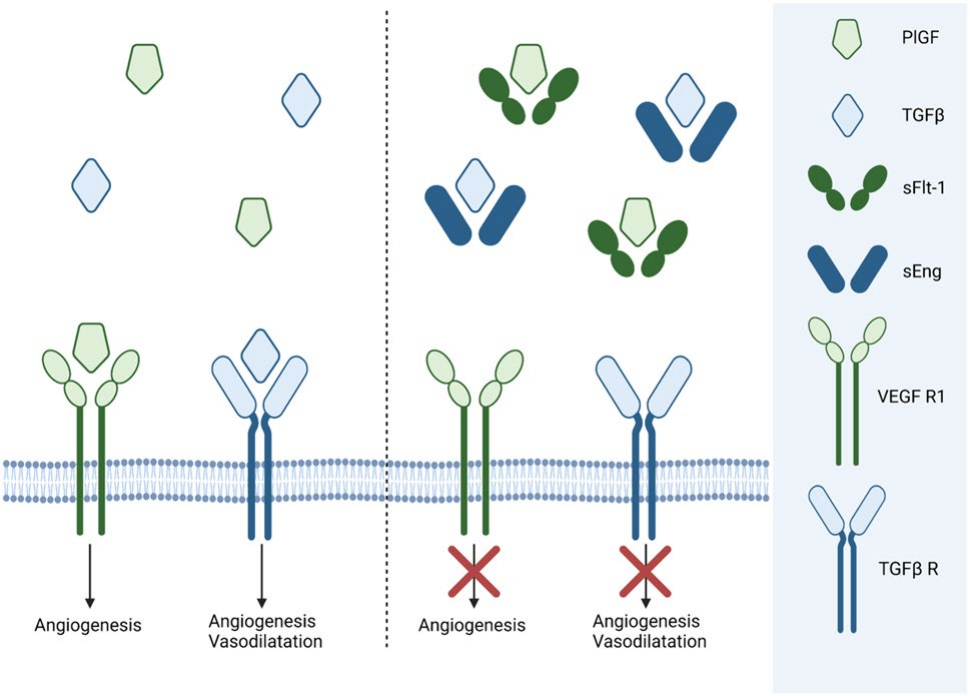
Pro-and anti-angiogenic factors that impact maternal vascular adaptation to pregnancy. PlGF stimulates angiogenesis in the utero-placental and feto-placental circulations by binding to the VEGF R1. TGFβ stimulates vasodilatation and angiogenesis via binding to the TGFβ R. sFlt-1, a soluble variant of VEGFR-1, binds to circulating PlGF thereby suppressing PlGF bioavailability and consequently inhibiting its proangiogenic functions. Similar to sFlt-1, sEng binds and neutralizes TGFβ. *PlGF* Placenta Growth Factor, *TGFβ* transforming growth factor beta, *sFlt-1* soluble fms-like tyrosine kinase-1, *sEng* soluble endoglin, *VEGF R1* vascular endothelial growth factor receptor-1, *TGFβ R* transforming growth factor beta receptor. This image was created in BioRender.com

The placenta produces pro- and anti-angiogenic factors that impact maternal vascular adaptation to pregnancy, including placental growth factor (PlGF), soluble fms-like tyrosine kinase (sFlt-1), and soluble endoglin (sEng) [6]. PlGF stimulates vasodilatation and angiogenesis in the utero-placental and feto-placental circulations by binding to the vascular endothelial growth factor receptor-1 (VEGFR-1) [6]. sFlt-1, a soluble variant of VEGFR-1, binds to circulating PlGF, thereby suppressing PlGF bioavailability and consequently inhibiting its proangiogenic functions [7]. Similar to sFlt-1, sEng binds and neutralizes free transforming growth factor beta (TGFβ), another molecule involved in vasorelaxation and angiogenesis (Fig. 1) [8, 9]. Circulating levels of these angiogenic factors are directly linked to vascular development and an imbalance in the second and third trimester plays an essential role in the pathogenesis of preeclampsia [7–11]. Yet, how circulating angiogenic factors relate to utero-placental vascular development in the first trimester remains to be fully established.

We recently proposed the utero-placental vascular volume (uPVV) and utero-placental vascular skeleton (uPVS) as non-invasive in-vivo imaging markers of first-trimester utero-placental vascular volumetric and morphologic development, using 3-dimensional (3D) power Doppler ultrasound, virtual reality (VR) and skeletonization [12, 13]. Increased absolute volumetric and morphologic development of the utero-placental vasculature might be favorable for pregnancy outcomes, as previous studies demonstrated that the uPVV and uPVS associate positively with embryonic and fetal growth and birth weight percentiles [14, 15]. Moreover, increased uPVS development is associated with lower incidence of placenta-related complications, including FGR, PE, and PTB [12]. Further, increased density of vascular branching is considered unfavorable as it is associated with decreased embryonic and fetal growth and an increased incidence of placenta-related complications [12, 14]. Therefore, we postulate that the uPVV and uPVS prove valuable to investigate associations between circulating angiogenic factors and utero-placental vascular development in the first trimester of pregnancy.

The aim of this study was to investigate whether increased levels of PlGF, decreased levels sFlt-1 and sEng, or decreased sFlt-1/PlGF and sEng/PlGF ratios are associated with increased development of the first-trimester utero-placental vasculature, measured with the uPVV and uPVS. Secondly, we investigated whether these parameters associate with placental volume (PV) as a measure of trophoblast tissue, and whether the associations are influenced by conception method, fetal sex and the occurrence of placenta-related complications.

## Methods

### Study design

We used data form the VIRTUAL Placenta cohort, embedded in the ongoing prospective Rotterdam Periconception Cohort (Predict Study) [16, 17]. Women were enrolled from a tertiary referral hospital between January 2017 and March 2018, if they were at least 18 years old, carried a singleton pregnancy of less than 10 weeks gestational age (GA), and gave written informed consent. Naturally conceived pregnancies as well as pregnancies achieved via in vitro fertilization (IVF) with or without intracytoplasmic sperm injection (ICSI) were eligible for inclusion. Women were excluded from analysis in case of oocyte donation and/or miscarriage. Upon inclusion, participants were required to complete a questionnaire detailing their general characteristics, medical and obstetrical history and lifestyle behaviors. Geographical origin was defined by country of birth according to the classification of Statistics Netherlands (CBS) as ‘Netherlands, ‘Europe’ and ‘Non-Europe’ [18].

Each participant visited at least twice in the first trimester at 7, 9 and 11 weeks GA, during which transvaginal 3D PD ultrasound scans of the whole gestational sac including the placenta and utero-placental vasculature were acquired using a GE Voluson E8 (GE, Zipf, Austria). The standardized ultrasound settings used were previously described (quality: max; pulse repetition frequency (PRF): 0.6; wall motion filter (WMF): low1; compound resolution imaging (CRI): off; power Doppler (PD) gain: − 8.0) [13]. Ultrasound examinations were performed according to international guidelines on safe use of Doppler ultrasound in the first trimester of pregnancy (ALARA-principle) [19].

Body mass index (BMI) was determined during the first study visit according to protocol for the measurements of height and weight. Pregnancy outcome data was retrieved from from medical delivery records.

At 11 weeks GA, maternal venous blood samples were drawn according to the study protocol [16, 17].

### Pregnancy dating

For naturally conceived pregnancies in regular cycles with a duration between 25 and 35 days, GA was calculated from the first day of last menstrual period (LMP). In case of unknown LMP or irregular cycle, GA was calculated from the Crown-Rump-Length (CRL). If the two methods varied > 6 days, the CRL-based GA was assumed the true GA. For fresh IVF/ICSI conceived pregnancies, GA was calculated from oocyte pick-up day + 14 days. In case of cryopreserved embryo transfer, GA was calculated from transfer date + 19 days.

### Definitions of placenta-related complications

Placenta-related complications were specified as pregnancy induced hypertension (PIH), PE and/or FGR, small-for-gestational-age (SGA) or PTB. PIH was defined as new-onset hypertension after 20 weeks GA with systolic blood pressure ≥ 140 mmHg and/or diastolic blood pressure ≥ 90 mmHg [20]. PE was defined as PIH with presence of ≥ 300 mg proteinuria in a 24-h period [20]. FGR was defined as a fetal abdominal circumference and/or EFW < 10th percentile on the Hadlock curve, or > 20 percentile decrease compared to a previous measurement with a minimal timespan of two weeks [21]. SGA was defined as birth weight < 10th percentile on growth curves specific for GA at birth, parity and fetal sex [22]. PTB was defined as a GA at birth < 37 + 0 weeks [23].

### Imaging markers of the first-trimester utero-placental vascular development

**Fig. 2:**
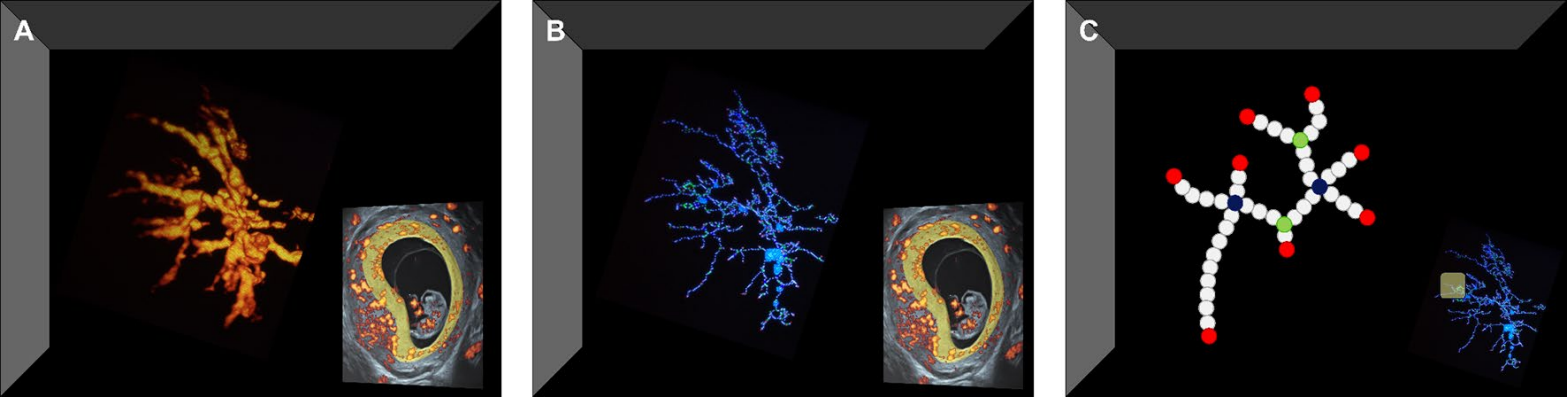
Overview of utero-placental (vascular) imaging markers; the placental volume (PV), utero-placental vascular volume (uPVV) and utero-placental vascular skeleton (uPVS) of a first-trimester pregnancy. **A**: Utero-placental vascular volume (uPVV) and slice view of a three-dimensional (3D) power Doppler (PD) ultrasound of a first trimester pregnancy (bottom right). The yellow-highlighted section in the bottom right image indicates placental tissue, which is confined by the placental-myometrial interface on the outside and the gestational sac on the inside. These anatomical boundaries are used to calculate the placental volume (PV). Using virtual reality-based segmentation, all PD signals outside the PV-segment were erased. The uPVV is calculated by summation of the thresholded 3D PD voxels (3D pixels) within the PV-segment. **B**: Utero-placental vascular skeleton (uPVS) and slice view of a three-dimensional (3D) power Doppler (PD) ultrasound of a first trimester pregnancy (bottom right). A skeletonization algorithm has been applied to the uPVV (from panel A), resulting in the uPVS. The skeletonization algorithm repeatedly peels off the outermost layer of voxels from the uPVV, reducing the diameter of the PD signal at each point in the vascular network until one central voxel remains, thereby creating a network-like structure representing the vascular morphology. **C**: A magnified portion of the uPVS and overview of the uPVS including the magnified portion (yellow-highlighted section) at the bottom right. Each voxel of the uPVS is automatically assigned a morphologic characteristic based on the number of adjacent voxels: red = endpoint (1 adjacent voxel); white = vessel point (2 adjacent voxels); green = bifurcation point (3 adjacent voxels); blue = crossing point (4 adjacent voxels). Two other characteristics are derived from the uPVS: 1. Total network length was calculated by summation of the total number of voxels in the skeleton, multiplied by 1 voxel length (mm). 2. Average vessel thickness was calculated by the average number of voxel-layers that were peeled off from the uPVV to reach the central voxel of the uPVS, multiplied by 1 voxel length (mm)

Image quality was assessed on a four-point scale ranging between zero (optimal) and three (unusable) based on the presence of artefacts, the ability to discriminate between myometrium and trophoblastic tissue, and completeness of the placenta. Images with a quality score of three were excluded from the analyses. The placental volume (PV in cm^3^) was measured using VOCAL software according to the previously published study protocol [24]. The utero-placental vascular volume (uPVV in cm^3^) was measured using a virtual reality (VR) desktop system with the V-Scope volume rendering application according to the previously published study protocol [13]. Figure 2A depicts the uPVV.

The utero-placental vascular skeleton (uPVS) was generated by applying a skeletonization algorithm to the uPVV segmentation [12]. According to the previously published study protocol, the skeletonization algorithm repeatedly peels off the outermost layer of voxels from the uPVV, reducing the diameter of the PD signal at each point in the vascular network until one central voxel remains, thereby creating a network-like structure representing the vascular morphology. The uPVS is depicted in Fig. 2B. Following the construction of the network, the skeletonization algorithm generates 6 uPVS characteristics to represent absolute morphologic development of the utero-placental vasculature: endpoints (n), bifurcation points (n), crossing points (n), normal vessel points (n), total vascular length (mm) and average vascular thickness (mm), which is illustrated in Fig. 2C. Finally, we calculated ratios of the uPVS end-, bifurcation- and crossing points to the uPVV as to identify 3 imaging markers representing density of vascular branching in the utero-placental vascular volume. Women who had no PV, uPVV or uPVS measurement available, were excluded from analysis.

Given the small contribution of embryonic vascular structures to the VR-based segmentation and the restricted presence of embryonic flow in the first trimester, we assume the contribution of the embryonic blood space to the uPVV and the uPVS is limited. Therefore, we conclude that the uPVV and uPVS mainly include the distal segments of the maternal spiral and basal arteries and communicating anastomoses (arteriovenous shunts) [12, 13].

### Assessment of PlGF, sFlt-1 and sEng in maternal serum

Directly after collection, all maternal serum samples were centrifuged and stored at − 80 °C until analysis. PlGF (pg/mL) and sFlt-1 (pg/mL) were measured using commercially available assays on the Elecsys platform (Roche Diagnostics), as previously described [25]. An enzyme-linked immunosorbent assay (ELISA) was performed for the determination of sEng (ng/mL) using the Quantikine® Elisa Human Endoglin/CD 105 commercial kit (R & D Systems, Abingdon, UK). For each sample, the sFlt-1/PlGF ratio and sEng/PlGF ratio were calculated [7, 26].

### Statistical analysis

Baseline characteristics are presented as mean and standard deviation or median and interquartile range (IQR). If needed, non-volumetric parameters were transformed using a square root transformation to approximate a normal distribution. For volumetric parameters and ratios a cubic root and natural log transformation were used respectively.

We performed linear mixed models to estimate the associations between maternal serum biomarkers and their ratios at 11 weeks GA (PlGF, sFlt-1, sEng, sFlt-1/PlGF ratio and sEng/PlGF ratio) and longitudinal measurements of the imaging markers of utero-placental vascular development (PV, uPVV and uPVS), using a quadratic relation with GA. We constructed model 1 (adjusted for GA) and model 2 (additionally adjusted for maternal age, BMI, parity, conception mode, fetal sex and periconceptional alcohol consumption, smoking and folic acid supplement use). These possible confounders were selected using a covariate-correlation matrix and supplemented with covariates based literature after discussion amongst authors.

All analyses were performed using SPSS (version 25.0; SPSS Inc., Chicago, IL, USA) and R (version 4.0.2, R Core Team, Vienna, Austria, 2020), p-values < 0.05 were considered statistically significant.

## Results

### Study population

**Fig. 3:**
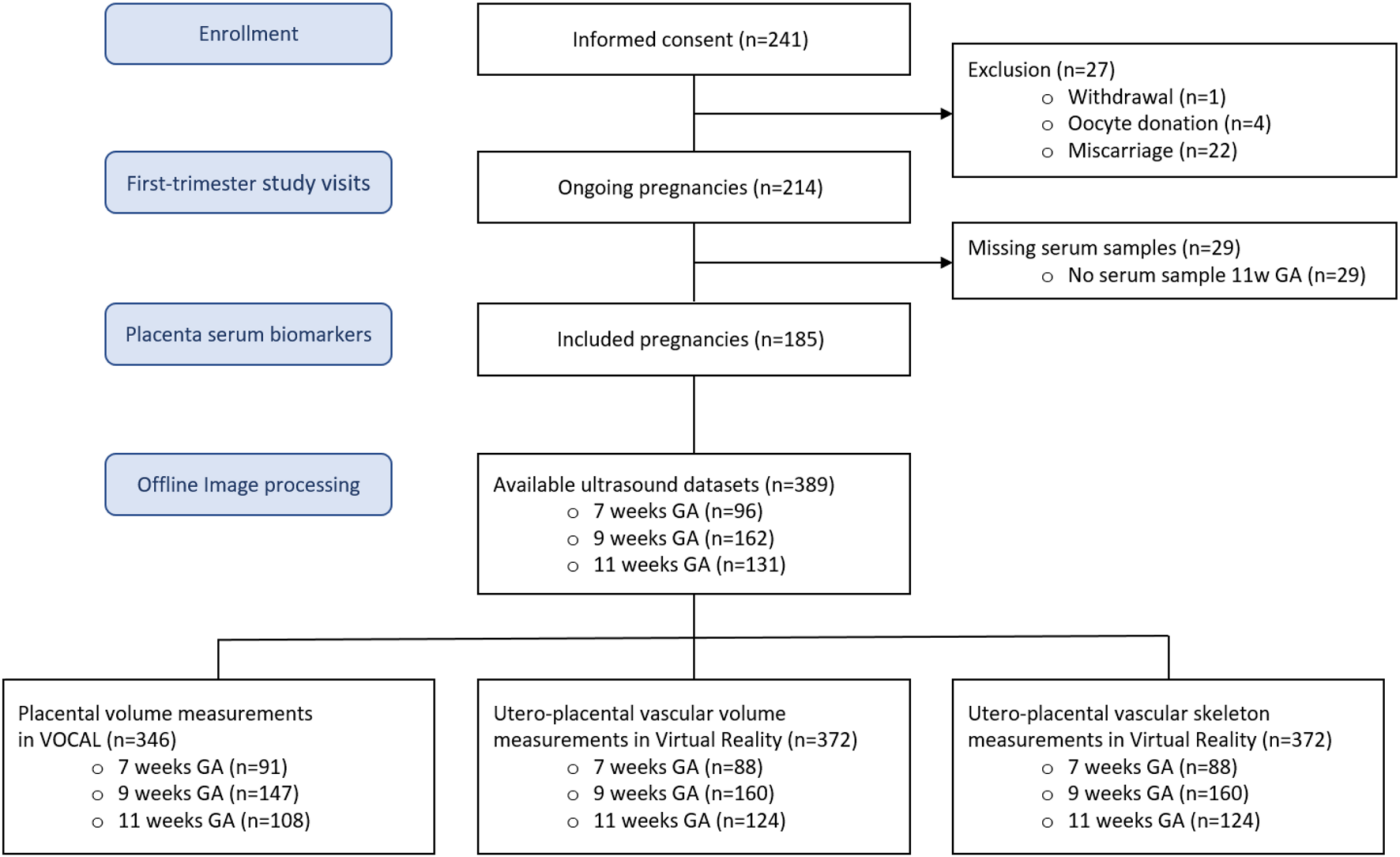
Flowchart of participant selection

A total of 241 women were enrolled in the VIRTUAL Placenta study. A flow chart of the study population is depicted in Fig. 3. We excluded 56 women from the analyses because of withdrawal (n = 1), miscarriage (n = 22), oocyte donation (n = 4), or missing maternal blood samples (n = 29). Finally, 185 women were included in this study. For each participant at least one PV or uPVV measurement was available. Table 1 shows the baseline characteristics of the study population. Participants were on average 32.3 years of age, 57.8% was nulliparous and 57.8% of pregnancies was achieved via IVF-ICSI. Table 2 summarizes serum concentrations of PlGF, sFlt-1 and sEng and the sFlt-1/PlGF and sEng/PlGF ratios and reference values obtained from the literature. Maternal serum concentrations of PlGF and sFlt-1 were significantly higher in pregnancies without placenta-related complications than in pregnancies with placenta-related complications (PlGF: Median 40.6 pg/mL (IQR 19.4) vs Median 36.3 pg/mL (IQR 17.5), p-value = 0.028; sFlt-1: Median 1337.0 pg/mL (IQR 485) vs Median 1082.5 pg/mL (IQR 400), p-value < 0.001). No statistically significant differences were observed for sEng, and the sFlt-1/PlGF or sEng/PlGF ratios between the two groups.

**Table 1:** Baseline characteristics of study population (n = 185)

	Total cohort (n = 185) Mean (SD) or n (%)
Maternal characteristics	
Age (years)	32.3 (4.4)
Nulliparous (yes)	107 (57.8%)
IVF/ICSI (yes)	107 (57.8%)
Geographic origin	
Netherlands	158 (85.3%)
Europe	4 (2.2%)
Non-Europe	19 (10.3%)
Missing	4 (2.2%)
Educational Level	
Low	16 (8.6%)
Intermediate	61 (33.0%)
High	105 (56.8%)
Missing	3 (1.6%)
BMI at first visit, (kg/m2)	26.0 (5.2)
Folic acid supplementation (yes)	155 (83.8%)
Alcohol consumption (yes)	52 (28.1%)
Smoking (yes)	27 (14.6%)
Neonatal outcomes	
Fetal sex (boys)	91 (49.2%)
GA at birth (weeks + days)	38 + 5 (20 days)
Birth weight (grams)	3233 (850)
Hoftiezer percentiles	46 (29)
Congenital anomalies	2 (1.1%)
Placenta-related complications^a^	46 (24.9%)
PIH^b^	8 (4.3%)
PE^b^	7 (3.8%)
FGR^b^	12 (6.5%)
SGAa^b^	17 (9.2%)
PTB^b^	18 9.7%)

^a^Placenta-related complications are specified as PIH or PE and/or FGR, PTB and SGA

^b^Diagnoses may be overlapping

*IVF* in vitro fertilization, *ICSI* intracytoplasmic sperm injection, *BMI* body-mass index, *GA* gestational age, *PIH* pregnancy induced hypertension, *PE* preeclampsia, *FGR* fetal growth restriction, *SGA* small-for-gestational-age, *PTB* preterm birth

**Table 2:** Maternal serum biomarkers at 11 weeks gestational age in the total cohort and stratified for pregnancies with and without a placenta-related complication (n = 185)

	Total cohort (n = 185) Mean (SD)	Without (n = 139) Mean (SD)	With (n = 46) Mean (SD)	p-value	Total Reference value Mean range	Without Reference value Mean range	With Reference value Mean range
PlGF (pg/ml)	41.6 (17.4) *Missing 0*	**43.1 (18.2)**	**36.9 (13.6)**	0.035*	35.4–62.8 [6, 27, 28]	35.4–144.0 [29–31]	26.2–153.0 [29–31]
sFlt-1 (pg/ml)	1332.6 (489) *Missing 0*	**1407.1 (509)**	**1107.7 (339)**	< 0.001*	973–1445 [6, 28]	1221–1788 [29–31]	915–1764 [29–31]
sEng (ng/ml)^a^	8.6 (2.8) *Missing 2*	8.6 (3.0)	8.6 (2.3)	0.997	8.2 [6]	5.0–7.2 [29, 30]	5.6–7.3 [29, 30]
sFlt-1/PlGF ratio	36.0 (17.8) *Missing 0*	36.9 (18.8)	33.3 (14.2)	0.244	22.7 [28]	32.9–117.0 [29, 31]	26.5–135.0 [29, 31]
sEng/PlGF ratio	239.9 (126.6) *Missing 2*	231.6 (125.5)	264.4 (128.1)	0.129	–	–	–

Placenta-related complications are specified as PIH or PE and/or FGR, PTB and SGA. Dashes represent the absence of published data quantifying protein levels. *PlGF* placenta growth factor, *sFlt-1* soluble fms-like tyrosine kinase-1, *sEng* soluble endoglin, *PIH* pregnancy induced hypertension, *PE* preeclampsia, *FGR* fetal growth restriction, *SGA* small-for-gestational-age, *PTB* Preterm birth. Student’s T-test. Bold values indicate statistically significant differences between the subgroups with and without placenta-related complications

^a^sEng is missing for two cases. In both cases the maternal serum concentration was below the detectable threshold of < 0.07 ng/ml and could therefore not be accurately determined

*Significance at p-value < 0.05

### PlGF, sFlt-1, sEng

Table 3 shows positive associations between maternal serum PlGF at 11 weeks GA and trajectories of PV (β = 0.53, 95% CI = 0.34; 0.73), uPVV, (β = 0.39, 95% CI = 0.15; 0.64) and uPVS (bifurcation points: β = 4.64, 95% CI = 0.04; 9.25; crossing points: β = 4.01, 95% CI = 0.65; 7.37; total vascular length: β = 13.33, 95% CI = 3.09; 23.58; average thickness: β = 0.05, 95% CI = 0.01; 0.08) and negative associations with density of vascular branching (end points: β = − 1.73, 95%CI = − 3.19; − 0.28; bifurcation points: β = − 0.76, 95%CI = − 1.38; − 0.14), all p-values < 0.05. In summary, higher maternal serum PlGF at 11 weeks is associated with both increased placental volume and absolute volumetric and morphologic (branching) development of the utero-placental vasculature and decreased density of vascular branching in the first trimester. We observed no associations between sFlt-1 and sEng and first-trimester development of PV, uPVV, uPVS characteristics or density of vascular branching, see Table 3.

**Table 3:** The association between maternal serum biomarkers of placental angiogenesis at 11 weeks GA and imaging markers of first-trimester utero-placental vascular development (n = 185)

	Model 2			Model 2			Model 2		
	*β*	95% CI	p-value	*β*	95% CI	p-value	*β*	95% CI	p-value
	$log$ PLGF (pg/ml)			$log$ sFlt-1 (pg/ml)			sEng (ng/ml)		
$\sqrt[3]{}$ PV (cm^3^)	**0.53**	**0.34; 0.73**	< 0.001*	0.19	− 0.08; 0.46	0.165	0.02	− 0.01; 0.05	0.169
$\sqrt[3]{}$ uPVV (cm^3^)	**0.39**	**0.15; 0.64**	0.003*	0.24	− 0.06; 0.54	0.114	− 0.02	− 0.05; 0.02	0.319
$\surd$ uPVS end points (n)	3.66	− 0.16; 7.48	0.061	0.69	− 3.78; 5.17	0.759	− 0.16	− 0.71; 0.38	0.548
$\surd$ uPVS bifurcation points (n)	**4.64**	**0.04; 9.25**	0.048*	2.05	− 3.34; 7.43	0.453	− 0.41	− 1.06; 0.24	0.215
$\surd$ uPVS crossing points (n)	**4.01**	**0.65; 7.37**	0.020*	1.95	− 2.00; 5.90	0.330	− 0.16	− 0.64; 0.32	0.505
$\surd$ uPVS vessel points (n)	9.82	− 0.75; 20.39	0.068	4.04	− 8.30; 16.38	0.518	− 1.02	− 2.44; 0.39	0.176
$\surd$ uPVS total length (mm)	**13.33**	**3.09; 23.58**	0.011*	6.58	− 5.51; 18.67	0.283	− 0.73	− 2.19; − 0.74	0.329
$\surd$ uPVS average thickness (mm)	**0.05**	**0.01; 0.08**	0.007*	0.04	− 0.00; 0.08	0.065	− 0.03	− 0.06; 0.02	0.319
$log$ density of end points (n/cm^3^)	**− 1.73**	**− 3.19; − 0.28**	0.020*	**− **1.328	**− **3.02; 0.38	0.126	0.09	**− **0.12; 0.29	0.419
$log$ density of bifurcation points (n/cm^3^)	**− 0.76**	**− 1.38; − 0.14**	0.017*	**− **0.56	**− **1.29; 0.17	0.129	0.01	**− **0.08; 0.10	0.811
$log$ density of crossing points (n/cm^3^)	**− **0.17	**− **0.56; 0.22	0.395	**− **0.21	**− **0.66; 0.24	0.364	0.04	**− **0.02; 0.09	0.173

Model 2: adjusted for gestational age, maternal BMI, parity, conception mode, fetal sex and periconceptional smoking, alcohol consumption and folic acid supplement use. *PLGF* placental growth factor, *sFlt-1* soluble fms-like tyrosine kinase-1, *sEng* soluble endoglin, *PV* placental volume, *uPVV* utero-placental vascular volume, *uPVS* utero-placental vascular skeleton

Bold values indicate statistically significant association between maternal serum biomarker concentration (ratio) at 11 weeks and first-trimester utero-placental vascular development

*significance at p-value < 0.05

### sFlt-1/PlGF ratio, sEng/PlGF ratio

Table 4 shows negative associations between the sFlt-1/PlGF ratio and the trajectory of PV (β = − 0.35, 95% CI = − 0.53; − 0.16, p-value < 0.001), but not with any of the 3D PD imaging markers of utero-placental vascular development. We observed negative associations between the sEng/PlGF ratio and trajectories of PV (β = − 0.26, 95% CI = − 0.44; − 0.08), uPVV, (β = − 0.30, 95% CI = − 0.52; − 0.09) and uPVS (bifurcation points: β = − 4.08, 95% CI = − 7.87; − 0.30; crossing points: β = − 2.80, 95% CI = − 5.59; − 0.01; vessel points: β = − 9.20, 95% CI = − 17.89; − 0.53; total vascular length: β = − 10.03, 95% CI = − 18.50; − 1.56; average thickness: β = − 0.03, 95% CI = − 0.06; − 0.01) and positive associations with density of vascular branching (end points: β = 1.38, 95% CI = 0.18; 2.58; bifurcation points: β = 0.54, 95% CI = 0.03; 1.06), all p-values < 0.05, see Table 4. To summarize, higher maternal serum sFlt-1/PlGF ratio at 11 weeks is associated with decreased first-trimester placental volume but not with vascular development. Higher maternal serum sEng/PlGF ratio is associated with both decreased placental volume and absolute volumetric and morphologic (branching) development of the utero-placental vasculature and increased density of vascular branching in the first trimester.

**Table 4:** The association between maternal serum biomarkers of placental angiogenesis at 11 weeks GA and imaging markers of first-trimester utero-placental vascular development (n = 185)

	Model 2			Model 2		
	*β*	95% CI	p-value	*β*	95% CI	p-value
	$log$ s-Flt-1/PLGF ratio			$log$ sEng/PLGF ratio		
$\sqrt[3]{}$ PV (cm^3^)	**− 0.35**	**− 0.53; − 0.16**	< 0.001*	**− 0.26**	**− 0.44; − 0.08**	0.005*
$\sqrt[3]{}$ uPVV (cm^3^)	**− **0.16	**− **0.40; 0.07	0.165	**− 0.30**	**− 0.52; − 0.09**	0.005*
$\surd$ uPVS end points (n)	**− **2.45	**− **5.85; 0.96	0.157	**− **2.45	**− **5.62; 0.715	0.128
$\surd$ uPVS bifurcation points (n)	**− **2.42	**− **6.53; 1.70	0.247	**− 4.08**	**− 7.87; − 0.30**	0.035*
$\surd$ uPVS crossing points (n)	**− **1.98	**− **5.00; 1.04	0.197	**− 2.80**	**− 5.59; − 0.01**	0.049*
$\surd$ uPVS vessel points (n)	**− **5.28	**− **14.71; 4.14	0.269	**− 9.20**	**− 17.89; − 0.53**	0.038*
$\surd$ uPVS total length (mm)	**− **6.52	**− **15.76; 2.71	0.164	**− 10.03**	**− 18.50; − 1.56**	0.021*
$\surd$ uPVS average thickness (mm)	**− **0.01	**− **0.04; 0.02	0.352	**− 0.03**	**− 0.06; − 0.01**	0.018*
$log$ density of end points (n/cm^3^)	0.57	**− **0.74; 1.89	0.388	**1.38**	**0.18; 2.58**	0.024*
$log$ density of bifurcation points (n/cm^3^)	0.26	**− **0.30; 0.83	0.353	**0.54**	**0.03; 1.06**	0.040*
$log$ density of crossing points (n/cm^3^)	0.01	**− **0.34; 0.36	0.956	0.29	**− **0.02; 0.61	0.070

Model 2: adjusted for gestational age, maternal BMI, parity, conception mode, fetal sex and periconceptional smoking, alcohol consumption and folic acid supplement use. *PLGF* placental growth factor, *sFlt-1* soluble fms-like tyrosine kinase-1, *sEng* soluble endoglin, *PV* placental volume, *uPVV* utero-placental vascular volume, *uPVS* utero-placental vascular skeleton

Bold values indicate statistically significant association between maternal serum biomarker concentration (ratio) at 11 weeks and first-trimester utero-placental vascular development

*significance at p-value < 0.05

### Stratification for conception method, fetal sex and the occurrence of placenta-related complications

**Fig. 4:**
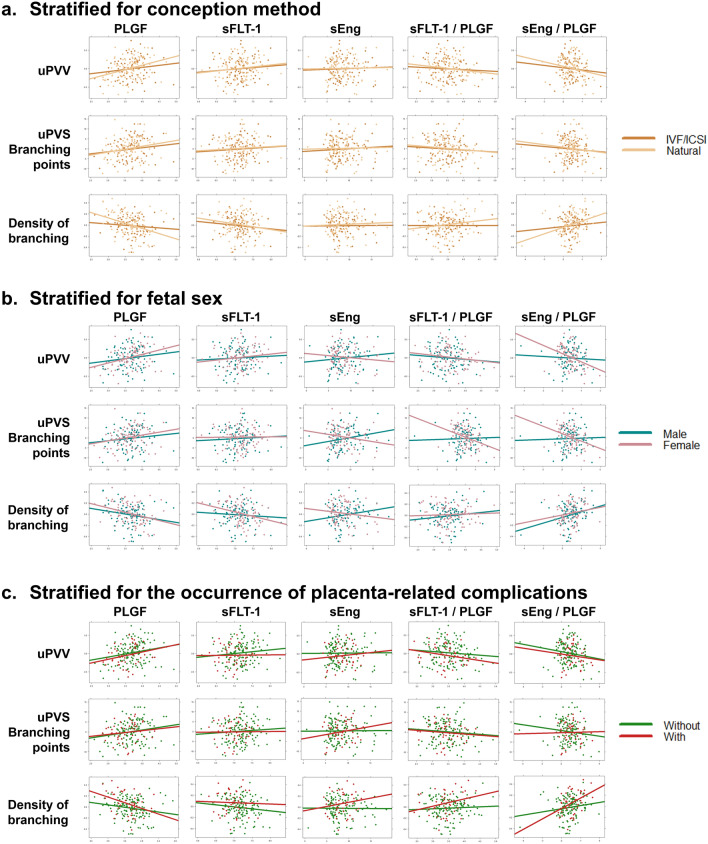
Correlation plot of maternal serum biomarkers at 11 weeks GA and imaging markers of the first-trimester utero-placental vascular development stratified for conception method, fetal sex and the occurrence of placenta-related complications. Unadjusted correlation plot. a. Stratified for conception method. b. Stratified for fetal sex. c. Stratified for the occurrence of placenta-related complications. Placenta-related complications are specified as PIH or PE and/or FGR, PTB and SGA. There are no statistically significant differences between the stratified groups. *uPVV* utero-placental vascular volume, *uPVS* utero-placental vascular skeleton, *PlGF* placenta growth factor, *sFlt-1* soluble fms-like tyrosine kinase-1, *sEng* soluble endoglin, *PIH* pregnancy induced hypertension, *PE* preeclampsia, *FGR* fetal growth restriction, *SGA* small-for-gestational-age, *PTB* preterm birth

Figure 4 depicts correlation plots of the maternal serum biomarkers and their ratios at 11 weeks GA and imaging markers of the first-trimester utero-placental vascular development stratified for conception method, fetal sex and the occurrence of placenta-related complications. There are no statistically significant differences between the subgroups.

## Discussion

Adequate utero-placental vascular development in the first trimester of pregnancy is essential to ensure optimal placental development and function, and consequently to achieve a healthy pregnancy outcome. In this study, increased maternal serum PlGF at 11 weeks is associated with increased first-trimester PV development, reflecting trophoblast tissue development and increased uPVV and uPVS development, reflecting volumetric and morphologic (branching) development of the utero-placental vasculature, respectively. Moreover, increased maternal serum sEng/PlGF ratio is associated with decreased first-trimester PV and decreased uPVV and uPVS development. These associations are not affected by conception method, fetal sex or the occurrence of placenta-related complications.

The positive association between maternal serum PlGF at 11 weeks and the novel uPVV and uPVS imaging markers substantiates that uPVV and uPVS measurements reflect development of the utero-placental vasculature in the first trimester of pregnancy.

Interestingly, our study revealed that the participants with an uneventful pregnancy had higher serum concentrations of sFlt-1 than participants that later experienced a placenta-related complication.

### Interpretation in light of other evidence

PlGF, a member of the vascular endothelial growth factor (VEGF)-family, stimulates angiogenesis and vasodilatation not only in the placenta, but also in other organs outside of gestation [32]. Animal and in vitro studies show PlGF is involved with branching angiogenesis (blood vessel formation) as well as non-branching angiogenesis (blood vessel elongation and enlargement) and plays an important role in the ability to employ compensatory mechanisms in pathologic conditions, such as ischemic tissue damage and tumor vascularization [32–36]. PlGF knockout mice exhibit aberrant utero-placental vascular development at gestational day 8.5, which corresponds to the late first-trimester in human pregnancy, including reduced branching [34]. Our study is the first to show the associations between circulating PlGF levels on first-trimester utero-placental vascular volumetric and morphologic development in vivo in humans.

Notably, we observed the strongest associations between PlGF and PV development. The PV measurements include but are not limited to the utero-placental vasculature. In fact, the PV measurements represent the whole placenta, which consists mainly of trophoblast cells. It is well known that trophoblast cells produce PlGF, which might explain why the association between PlGF and PV is stronger than associations with the uPVV or uPVS [37].

We did not observe any associations between sFlt-1, the soluble antiangiogenic receptor of PlGF, and first-trimester placental volume, utero-placental vascular volume or morphologic development. Although a few animal studies show increased early and mid-gestational levels of sFlt-1 are associated with impaired spiral artery remodeling [38, 39], these associations have not been studied in humans. One study found positive associations between sFlt-1 levels and the uterine artery pulsatility index, as an indirect measure of utero-placental vascular development, in the third trimester [40], but these associations were not observed in the first and second trimester [41, 42].

Studies comparing first-trimester sFlt-1 levels between healthy pregnancies and pregnancies with preeclampsia or fetal growth restriction report ambiguous results [43–46]. Importantly, clinically relevant rises of sFlt-1 are observed only from 5 weeks before the onset of preeclampsia [47]. As a consequence, elevated sFlt-1 levels are almost exclusively observed in the second and third trimester. Our study’s findings seem to confirm that circulating sFlt-1 does not have a substantial influence on utero-placental vascular development in the first trimester.

Remarkably, in our study we observed lower sFlt-1 serum concentrations in pregnancies that later developed a placenta-related complication compared to the group with an uneventful pregnancy, which is in line with previous findings by Schiffer et al. [31]. However, other studies found first-trimester sFlt-1 to be higher in pregnancies that later developed preeclampsia [29, 48]. These seemingly paradoxical results pose interesting conjecture for future research.

We observed no associations between sEng, a small receptor with anti-angiogenic properties by limiting the bioavailability of proangiogenic TGFβ, and first-trimester placental volume, utero-placental vascular volume or morphologic development. Although several studies have investigated the predictive value of sEng in the first trimester for the risk of preeclampsia or fetal growth restriction, no studies have researched its associations with utero-placental vascular development. We found one study that found a negative correlation between sEng levels and the uterine artery pulsatility index in the second trimester, but not in the first trimester, which is in accordance with our results [42].

In our study, there were no differences in sEng levels between women with and without placenta-related complications. Some studies show higher first-trimester levels of sEng in women ultimately developing preeclampsia [29, 49]. Notably, in our study, placenta-related complications comprised a heterogeneous group of clinical conditions defined as the presence of PIH, PE and/or FGR, SGA or PTB, which could explain why we observed no associations. Further, other studies show elevated sEng levels in pregnancies which later developed preeclampsia from 17 weeks onwards [50], which may also explain the lack of associations in our study.

We found no associations between the sFlt-1/PlGF ratio and utero-placental vascular development. Our results imply that, at least in the first trimester, free PlGF levels are relevant for physiological angiogenesis (utero-placental vascular development) irrespective of sFlt-1 levels. These findings are in line with previous research, which suggests free PlGF concentrations are diminished in patients with preeclampsia independent from sFlt-1 concentrations [51]. Our findings are further substantiated by a recent study that shows the imbalance of sFlt-1/PlGF ratio in preeclampsia is mainly caused by diminished PlGF production as opposed to increased sFlt-1 levels [52]. These findings give rise to the possibly that, in the first trimester, an increase of sFlt-1 in is accompanied by a proportional rise in PlGF, maintaining the concentration of free PlGF constant. In later pregnancy, during the late second and third trimester, excessive rises in sFlt-1 are no longer matched by PlGF, which leads to a sFlt-1/PlGF ratio imbalance and gives rise to placenta-related complications.

Although previous studies have found associations between the sEng/PlGF ratio and pregnancy outcomes [26], no studies have investigated its associations with angiogenesis. The sEng/PlGF ratio can be interpreted as a composite ratio reflecting both the VEGF- and TGFβ-pathways involved with angiogenesis. In our study, associations with PlGF are stronger than associations with the sEng/PlGF ratio. In addition, we found no associations with sEng. Therefore, the associations between the sEng/PlGF ratio and imaging markers of utero-placental vascular development are likely contributable to the effect of PlGF and we consider the possibility the TGFβ-pathways is involved with first-trimester utero-placental vascular development unconvincing.

### Strengths and limitations

One of the major strengths in our study is the use of validated test kits for the analyses of PlGF, sFlt-1 and sEng. Further, we made use of longitudinal first-trimester 3D power Doppler ultrasounds to perform validated 3D virtual reality based segmentations for the measurements of uPVV and uPVS [12, 13].

Unfortunately, all serum biomarker concentrations were determined only once during these pregnancies. Some studies show longitudinal changes in PlGF, sFlt-1 and sEng are potentially more clinically relevant than absolute levels [30, 53]. Longitudinal analysis may provide additional insights in the interplay between serum biomarker concentrations and first-trimester imaging markers of utero-placental vascular development.

The number of imaging markers used in this study might introduce some questions regarding the issue of multiple testing. However, our research has repeatedly demonstrated positive strong correlations between the uPVV and uPVS and an inverse association with density of vascular branching. Therefore, we argue the consistency of the presence or absence of these relationships can be viewed as internal validation of our findings.

### Implications and conclusion

This study’s findings suggest higher free PlGF concentrations likely contribute to increased first-trimester utero-placental vascular development as reflected by uPVV and uPVS, thereby providing new insights into potential mechanisms underlying placenta-related complications. Moreover, these results substantiate a role for PlGF in early prediction models for placenta-related complications. In contrast, sFlt-1 and sEng appear not to have a substantial influence on first-trimester utero-placental vascular development and therefore the added value of first-trimester sFlt-1 and sEng for early detection of placenta-related complications will be limited.

Importantly, the uPVV and uPVS can be used as 3D power Doppler imaging markers to monitor the volumetric and morphologic development of the utero-placental vasculature throughout the first trimester of pregnancy. Future application of the uPVV and uPVS measurements could benefit research on early vascular development to improve our understanding of the pathophysiology of placenta-related complications and the mechanisms underlying both therapeutic and preventative regimens, such as prophylactic administration of aspirin.

Finally, as maternal serum PlGF is currently used in multiple prediction models for preeclampsia, we recommend investigating the added value of the uPVV and uPVS as non-invasive and uncostly predictors for preeclampsia.

## Data Availability

Datasets generated and analyzed during the current study are not publicly available but are available from the corresponding author on reasonable request.
